# 

**Published:** 1893-07-15

**Authors:** 


					u. n::" r; ?? ? vv vrt.iivu.
Ho. 855, Vol. XIV.?July 15th, 1893.
(Batfatared at the General Post OflVoe aa a Newipaper (or ^ Ditto par Anirnin, 8a., post free.
?n?raugion in the United Kingdom and Abroad.]
price twopence. Ef fri
Monthly Parts. 6d.; poat free, 84.
' - 35g Vol.XIY. Saturday,
July 15th, 1893.
[TWOPENCE. 4

				

